# Characterization of new IS elements and studies of their dispersion in two subspecies of *Leifsonia xyli*

**DOI:** 10.1186/1471-2180-8-127

**Published:** 2008-07-25

**Authors:** Marcelo M Zerillo, Marie-Anne Van Sluys, Luis Eduardo A Camargo, Claudia B Monteiro-Vitorello

**Affiliations:** 1 Departamento de Botânica, Instituto de Biociências, Universidade de São Paulo, Rua do Matão, 277, 05508-900, São Paulo, SP, Brazil; 2 Departamento de Fitopatologia, Escola Superior de Agricultura Luiz de Queiroz, Universidade de São Paulo, Av. Pádua Dias, 11, 13418-900, Piracicaba, SP, Brazil; 3 Laboratório de Bioinformática, Laboratório Nacional de Computação Científica, Av. Getúlio Vargas, 333; Quitandinha, 25651-075, Petrópolis, RJ, Brazil; 4 Centro de Ciências Naturais e Humanas, Universidade Federal do ABC, Rua Santa Adélia, 166, 09210-170, Santo André, SP, Brazil

## Abstract

**Background:**

*Leifsonia xyli *is a xylem-inhabiting bacterial species comprised of two subspecies: *L. xyli *subsp. *xyli *(*Lxx*) and *L. xyli *subsp. *cynodontis *(*Lxc*). *Lxx *is the causal agent of ratoon stunting disease in sugarcane commercial fields and *Lxc *colonizes the xylem of several grasses causing either mild or no symptoms of disease. The completely sequenced genome of *Lxx *provided insights into its biology and pathogenicity. Since IS elements are largely reported as an important source of bacterial genome diversification and nothing is known about their role in chromosome architecture of *L. xyli*, a comparative analysis of *Lxc *and *Lxx *elements was performed.

**Results:**

Sample sequencing of *Lxc *genome and comparative analysis with *Lxx *complete DNA sequence revealed a variable number of IS transposable elements acting upon genomic diversity. A detailed characterization of *Lxc *IS elements and a comparative review with IS elements of *Lxx *are presented. Each genome showed a unique set of elements although related to same IS families when considering features such as similarity among transposases, inverted and direct repeats, and element size. Most of the *Lxc *and *Lxx *IS families assigned were reported to maintain transposition at low levels using translation regulatory mechanisms, consistent with our *in silico *analysis. Some of the IS elements were found associated with rearrangements and specific regions of each genome. Differences were also found in the effect of IS elements upon insertion, although none of the elements were preferentially associated with gene disruption. A survey of transposases among genomes of Actinobacteria showed no correlation between phylogenetic relatedness and distribution of IS families. By using Southern hybridization, we suggested that diversification of *Lxc *isolates is also mediated by insertion sequences in probably recent events.

**Conclusion:**

Collectively our data indicate that transposable elements are involved in genome diversification of *Lxc *and *Lxx*. The IS elements were probably acquired after the divergence of the two subspecies and are associated with genome organization and gene contents. In addition to enhancing understanding of IS element dynamics in general, these data will contribute to our ongoing comparative analyses aimed at understanding the biological differences of the *Lxc *and *Lxx*.

## Background

The Gram-positive, coryneform, fastidious, xylem-inhabiting bacteria *Leifsonia xyli *comprises two subspecies: *L. xyli *subsp. *xyli *(*Lxx*) and *L. xyli *subsp. *cynodontis *(*Lxc*). In its unique natural host, *Lxx *causes ratoon-stunting disease, a malady that affects sugarcane commercial fields worldwide, promoting losses of up to 30% in susceptible varieties [1]. Sequencing of the *Lxx *genome has provided important insights into the biology and pathogenicity of this bacterium [2]. *Lxc *is an endophyte of Bermuda grass (*Cynodo dactylon*) and, when artificially inoculated, can grow in and colonize the xylem of agriculturally important grasses (including sugarcane, corn and rice), causing no (or mild) symptoms of disease [3,4]. Some studies suggested that *Lxc *may be a potential vector for expressing heterologous proteins in plants [5-10,4]. We have initiated a genome-based approach to compare *Lxc *and *Lxx *by sample sequencing the *Lxc *genome. Our goal is to comprehensively assess gene content and genomic organization of these two closely related bacteria to enhance understanding of the differences in their pathogenicity and host range. Here, we present the *in silico *characterization of insertion sequence (IS) elements, the most abundant type of mobile genetic element found in *L. xyli *[2] and their involvement in *Lxc *and *Lxx *genome diversification.

IS elements are small transposable DNA fragments ranging from 0.7 to 3.5 kbp, comprising a transposase-encoding gene and terminal inverted repeats (IR) [11]. Close to 1,500 different IS elements have been reported in the chromosomes and plasmids of nearly all bacteria studied [12]. IS elements may inactivate genes upon insertion or activate and/or enhance the expression of nearby genes. Some are known to recognize specific sites of the genome that are duplicated after IS insertion, resulting in direct repeats (DR). IS elements may provide the structural basis necessary to enable the rearrangement of genomic fragments and the incorporation of foreign DNA either by active transposition process or indirectly, mediating homologous recombination between multiple copies present in a given genome [13]. They are believed to undergo frequent horizontal transfer and cycles of expansion and extinction within a given species, most likely as a consequence of transfer between genomes and plasmids [14]. Their expansion, genome location and composition may differ among related bacteria, representing an important source of genomic diversity [15-20,12]. Because its effects have a direct impact on cell survival, control of transposition is tightly regulated. Intrinsic regulation is basically at the transcriptional and translational level [21]. In addition, several host proteins have been identified as part of the transpososome, the assembly of which may be controlled by host factors, thus integrating transposition activity and host physiology [22].

Previously, 50 copies of five distinct IS elements (IS*Lxx*1–5) in the *Lxx *genome were reported, along with 47 other transposase-related genes from uncharacterized elements [2,23]. In *Lxc*, three IS elements have previously been identified: IS*1237 *[24], IS*Lxc*1 and IS*Lxc*2 [25], located both on the chromosome and on the cryptic plasmid pCXC100 that is present in some isolates. A detailed characterization of two new *Lxc *IS elements (IS*Lxc*3 and IS*Lxc*4) and one new *Lxx *element (IS*Lxx*6) is presented, as well as a comparative review of all elements found in both genomes, their distribution among families [11], and their comparative localization. The results support the hypothesis that IS elements are components involved in the diversification of *Lxx *and *Lxc*, with their influence most likely occurring after the divergence of the two subspecies from their common ancestor.

## Results

### The dataset of *Lxc *genomic DNA

The characterization of the IS elements were realized within the dataset of *Lxc *DSMZ46306 genomic DNA. The dataset is comprised of 9,766 reads, of which 5,854 were derived from the shotgun library and 3,912 from sequencing BAC ends, sub-cloning of inserts and primer-walking. All sequences were assembled into 1,064 contigs accounting for 1,368,731 non-redundant bases, representing approximately 50% of the *Lxc *chromosome. Comparing all the sequences of *Lxc *with the complete genome of *Lxx*, we found that nearly 70% share more than 80% nucleotide sequence identity considering a continuous segment of at least 200 bases. Two other genomes of related subspecies *Clavibacter michiganensis *subsp. *michiganensis *and *C*. *michiganensis *subsp. *sepedonicus *that were recently sequenced [26,27] have nearly 80% of nucleotide identity using the same criteria. These figures were derived from the Artemis Comparative Tool [28].

### *Lxc *and *Lxx *each have their own set of IS elements

Among all the contigs, 70 shared sequence similarity to transposase-encoding genes. Fifty-six of these represented copies of five distinct IS elements that were characterized based on the criteria proposed (Table 1). The remaining 14 contigs correspond to uncharacterized IS elements, since no IR, site of insertion and structural limits were identified within the available sequences. They were classified only tentatively based on homology searches with *Lxx *genome, and were placed within IS families (Table 2): IS*3 *family (eleven elements); IS*256 *family (two elements); and IS*481 *family (one element). Elements of the same families were not characterized in *Lxx *as well [2,23]. These uncharacterized transposases may represent degenerated forms of old insertions, however they may also be used as site for rearrangements within *Lxc *genome.

The transposases of characterized IS elements shared less than 78% amino acid sequence identity with transposases of *Lxx *elements (Additional file 1). To be considered as iso-forms, transposases of a given element must share more than 95% identical amino acids [11]; therefore, *Lxc *and *Lxx *have different sets of IS elements. Despite of that, most of the elements found belong to the same families (Table 2), with the exception of IS*110 *(IS*Lxx*2), which was not identified in our *Lxc *dataset.

**Table 1:** Details of *Lxc*-IS elements.

***Lxc*-IS**	**IS length (bp)**	**Tpase length (aa)^a^**	**IRs**	**DR (host sequence)**	**Minimal n^o ^of copies**	**IS family^b^**	**Most related *Lxx*-IS**
IS*1237*	899/798	218	5' GAGGTGGTTTCAGTAGTCG 3' GAGATGGTTTCAGTAGTCG^c^	TTA (TAA)	26	IS*5*	IS*Lxx*6
IS*Lxc*1	2631	262 (*ist*B)	5' TGTCTGTGTCACTGTTAGCGGGCTCCG GGTTGTCGGAGTTGTCTGGCCCCGGCG 3' TGACTATGTCAAGGCTGGCGGGGACCG GGTTGTCGAAGTTAGTTGGCCCCAGCG	fATCGCC	2	IS*21*	IS*Lxx*3
IS*Lxc*2	1105	332	5' TGTGTTTCTCAGGGACGTTGGT 3' TGTGCTGCTCAGGGACGTTGGT	NCTAGN	16	IS*481*	IS*Lxx*4
IS*Lxc*3	1511	351	5' CTTGGGTTCTAACGGTCGTTGCAACA 3' GGCGGATTCTAGTGGTCGTTGCAACA	-	8	IS*30*	IS*Lxx*5
IS*Lxc*4	1311/896	380	5' GGGTGGGTTCTAACAGTGGTCGCAACAC 3' GGGTGGGTTCTAACAGTGGTCGCAACAC	-	5	IS*30*	IS*Lxx*5

N – not complementary nucleotides

^a ^– assuming predicted features for regulation of transposition at translational level are overcome.

^b ^– according to Chandler & Mahillon family classification [11].

^c ^– three IS*1237 *copies have a G instead of A in the 3' IR, making perfectly complementary IRs.

**Table 2:** Classification of *Lxc *and *Lxx *transposase-encoding genes into IS families [11].

	*Lxc*			*Lxx*		
	
IS Family	characterized	non-characterized	**% Total**	characterized	non-characterized	**%Total**
*5*	26	0	**37**	1	4	**5**
*21*	1	0	**1**	5	3	**8**
*481*	16	1	**24**	26	6	**33**
*30*	13	0	**19**	15	0	**16**
*110*	0	0	**0**	4	1	**5**
*3*	0	11	**16**	0	19	**20**
*256*	0	2	**3**	0	8	**8**
undefined	0	0	**0**	0	5	**5**

Total	56	14	**100**	51	46	**100**

Columns in bold represent the percentage from the total number of predicted transposases in the *Lxx *genome and *Lxc *datasets. The other values are the numbers found within each genome.

### Description of *Lxc *IS elements and comparison with related elements in *Lxx*

**Figure 1:**
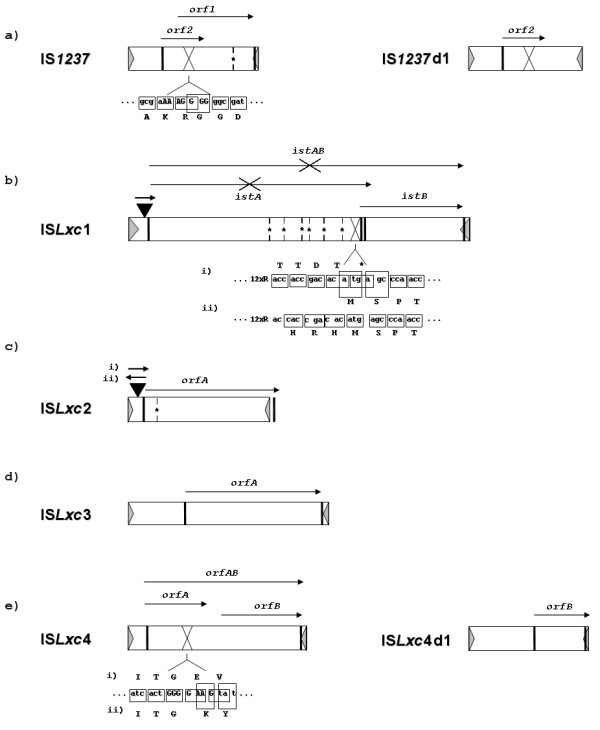
**Characterization of IS elements of *Lxc *dataset**. Each characterized IS element is represented by a rectangle, with IRs indicated by gray triangles. Black vertical bars indicate start and stop codons of the deduced transposases, and their transcriptional orientation is indicated by arrows. Crossed arrows represent truncated ORFs. Regions with slippery codons for frameshift are represented by X and the corresponding nucleotides are depicted in capital letters, with other nucleotides in lowercase letters. Amino acid sequences are shown beneath boxed codons. Vertical bars with asterisks represent premature stop codons. Inverted black triangles indicate the location of an IS*1237 *insertion within other IS elements, and the arrows above indicate the orientation of transcription of its transposase. In (b) we presented the nucleotides and deduced amino acid sequences immediately after the purine rich region that may promote either: (i) independent translation of istA and istB, or (ii) translation of istAB, depending on ribosomal slippage. In (c) copies of IS*1237 *were found inserted in both orientations (i and ii) when considering transcription of its transposase within IS*Lxc*2. (e) in (i) indicates independent translation of orfA and (ii) shows translation of the same nucleotides after a +1 frameshift (translation of orfAB).

In addition to the three IS elements previously sequenced for *Lxc*: IS*1237 *(GenBank ID: X75973); IS*Lxc*1 (GenBank ID: EF437436); and IS*Lxc*2 (GenBank ID: EF176596), we identified two new elements, named IS*Lxc*3 (GenBank ID: EF421582) and IS*Lxc*4 (GenBank ID: EF433175) (Table 1, Fig. 1 and Additional file 2). A detailed characterization of all these *Lxc*-IS elements, and a comparison with related elements in *Lxx*, is presented below.

#### *IS1237 *and IS*Lxx6*, IS*5 *family members

IS*1237 *was first identified in the plasmid pCXC100 detected in some *Lxc *isolates [24]. Recently, 13 upstream and 10 downstream flanking sequences of IS*1237 *were described [25]. We identified 26 copies of IS*1237 *(Additional files 3 and 4) represented by two variant forms: 24 copies were 899-bp long and almost identical, with a single polymorphism in the 3'-IR; two copies were 798 bp long (Table 1 and Fig. 1). Four copies were inserted within other IS elements: one inserted within IS*Lxc*1 and three inserted within copies of IS*Lxc*2. There was no read-through ORF encoding an entire transposase within IS*1237 *[24]. If functional, the coding sequence, starting and ending at nucleotide positions 232 and 889, respectively, would have to change frame (-1) and overcome a premature stop codon (Additional file 2). Since the coding sequence of all 899-bp long copies sequenced so far were identical, it is reasonable to assume that such translational features could be involved in the negative control of transposition [21]. The putative transposase has the expected catalytic domain containing a DDE motif [25], but the number of amino acids separating these residues does not follow the pattern of the IS*5 *family, and the glutamic acid is located downstream of the premature stop codon. Analyzing *Lxx *genome sequence, we identified IS*Lxx*6, a single copy of which is represented within the genomic island LxxGI3 [2]. In *Lxx*, LxxGI3, is a depository of IS elements encompassing seventeen putative transposase genes [2,23]. IS*Lxx*6 shows the highest nucleotide sequence similarity with an *Lxc*-IS element, the 899 bp-long IS*1237 *(Additional file 1). Interestingly, however, contrary to IS*1237*, the IS*Lxx*6 putative transposase gene (Lxx22320.1) is read-through (Additional file 5). Both elements were classified as belonging to group IS*427 *within IS*5 *family based on multiple alignment using Tribe-MCl (Patricia Siguier, personal communication). However, they share conserved amino acids with elements of group IS*1031 *(Additional file 5). In addition, IS*Lxx*6 presents a single ORF and recognize three nucleotides as site of insertion, also common features of elements of IS*1031 *group. This may indicate the existence of another group within IS*5 *family.

#### IS*Lxc*1, an IS*21 *family member

IS*Lxc*1 [25] encompasses two ORFs similar to the IstA and IstB transposases found in elements of the IS*21 *family [11]. In our *Lxc *dataset, we identified one copy of IS*Lxc*1, which was invaded by an *IS1237 *element. The IS*1237 *insertion is located 58 bp upstream of the first ORF (*ist*A) (figure 1) and has duplicated the "TAA" site of insertion within IS*Lxc*1 (Additional file 2). A second version of IS*Lxc*1 not carrying a copy of IS*1237 *was amplified in DSMZ46306 with inwardly oriented primers complementary to the 5' and 3' IRs. However this element was not mapped on *Lxx *genome because we did not have the flanking sequences. IS*Lxc*1 is 2,631 bp long and 99% identical to that previously sequenced [25]. Within both versions *istA *is truncated and the DDE motif was not identified, therefore *istA *disruption was prior to the IS*1237 *insertion. *istB *may encode a 262-amino-acid protein containing the predicted nucleoside triphosphate-binding domain. It is preceded by a purine reach site, which in other elements is an indicative of ribosome frameshifting and coupled translation of IstA and IstB [11] (Fig. 1 and additional file 2).

#### IS*Lxc*2, an IS*481 *family member

IS*Lxc*2 is a 1,105-bp-long element (Table 1 and Fig. 1) [25]. The 16 copies identified here all contain at least one nucleotide polymorphism in different positions, none of which located in crucial sites such as start and stop codons or the DDE motif. The putative transposase contains 332 amino acids and is interrupted by an in-frame stop-codon located 111 nucleotides downstream of the initiation codon (Additional file 2). Interestingly, the transposases of IS*Lxc*2 and also IS*Lxx*4 of *Lxx *genome [2] have stop codons within the host insertion site. The NCTAGN sequence is duplicated after element insertion and flanks both ends indicating that the translational end of the transposase may impose the structural limits on these elements, as described for other elements of the same family [29,30].

Three copies of IS*Lxc*2 were invaded by IS*1237*, in all the cases the insertion was located 13 bp upstream of the putative transposase gene, duplicating the TTA site of recognition. Generally, genes invaded by IS elements become non-functional, but the IS*1237 *insertions were all at the same position within IS*Lxc*2, located upstream of the transposase gene (Fig. 1). IS elements located upstream of the coding region may create a hybrid promoter between the IS element sequence and the gene and favor transcription of the later, which in this case is IS*Lxc*2 [11,21]. However, this should be addressed experimentally.

#### IS*Lxc*3 and IS*Lxc*4, IS*30 *family members

Two novel elements were identified: IS*Lxc*3 and IS*Lxc*4 (Table 1, Fig. 1 and Additional file 2). Eight copies of IS*Lxc*3 were found, six of which being 1,511 bp long, and two copies with 3' end deletions at different positions. One of these defective variants (occurrence number 8 – additional file 3) is associated with a reorganization event in *Lxc *compared to *Lxx *because one of the flanking regions is common to *Lxx *and the other one is specific to *Lxc*. This is an example showing that an IS element may serve as a subject for homologous recombination, independent of its integrity. An imperfect IR of IS*Lxc*3 was defined as a twenty-six bp long sequence starting with three non-complemented nucleotides, "CTT", at the 5'-end and "GCC" at the 3'-end, which were also detected for IS*Lxx*5 [2]. The proposed IRs were based on the experimentally proved structural limits of IS*1655*, an IS*30 *family element of *Neisseria meningitides *[31]. In fact, the first nine nucleotides of 3'-IR are conserved among IS*1655*, IS*Lxc*3 and IS*Lxx*5. We failed in detect a conserved target site for insertion in multiple alignment considering 20 bases upstream and downstream of the element limits, but the duplication of NTG sequence was detected flanking two copies of IS*Lxc*3. The putative transposase was the only one described in *Lxc *that was not interrupted by a premature stop codon or by a purine repeat associated with a frameshift.

IS*Lxc*4 was found in five copies represented by two variant forms. Two copies represented longer versions (1,311 bp) and the other three copies (896 bp) showed the same deletion within the core region, which were considered shorter versions of the IS*Lxc*4 (IS*Lxc*4d1 – GeneBank ID: EF494674). The IRs are twenty-eight bp long, and no DR was identified flanking the occurrences. Regardless of been unusual for IS*30 *family elements, the long version contain a cluster of purines (nucleotide position 431–437), resembling a slippery codon for the frameshift (Additional file 2). Transposases of the IS*30 *family encompass the helix-turn-helix (HTH) DNA-binding domain at the N-terminal part and the well-conserved D-(54–61 aa)-D-(33 aa)-E motif at the C-terminal part [30,32]. In IS*Lxc*4, if the +1 frameshift does occur, the transposase (OrfAB) is 380 amino acids long and contains two domains. If the frameshift does not occur, the transposase (OrfA) is 115 amino acids long, carrying only the DNA-binding domain. Truncated transposases with only the DNA-binding domain may function as negative regulators of transposition when interacting with IRs, resulting in either repression of the transposase gene promoter or competition with full-length transposases [33,21]. Neither the DNA-binding domain nor DDE motif following the rule above were detected within the ORF of the shorter variant.

### Analysis of IS element loci in the *Lxc *and *Lxx *genomes

**Figure 2:**
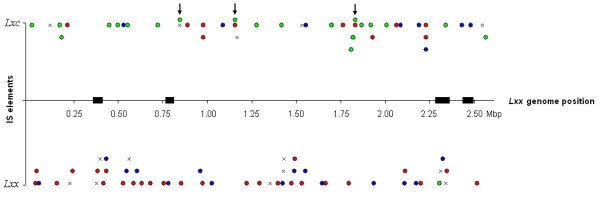
**Distribution of *Lxc *and *Lxx *IS elements across the complete nucleotide sequence of *Lxx *genome**. Mapping of *Lxc *elements was based on similarity of flanking sequences. Elements located less than 20 kbp apart are shown on different levels for ease of visualization. Elements sharing the same family assignment and possibly the same target site of insertion are represented by the same symbol in *Lxc *and *Lxx*: IS*1237 *and IS*Lxx*6 (green circles); IS*Lxc*2 and IS*Lxx*4 (red circles); IS*Lxc*3 and IS*Lxx*5 (blue circles). The remaining IS elements are marked as "X". Arrows indicate insertions of IS*1237 *copies within other IS elements. Black boxes indicate four genomic islands described for *Lxx *genome.

The average nucleotide sequence identity between homologous fragments of *Lxc *and *Lxx *is 93%, as calculated on 80 kbp of 25 continuous *Lxc*-contigs, which were physically linked based on scaffold orientation. Comparing the adjacent regions of all 56 *Lxc*-IS element with the *Lxx *sequence, 44 elements could be mapped onto the *Lxx *genome (Additional file 3). All the flanking sequences of IS elements were submitted to GenBank and the accession number is available in the additional file 3. Four of these (occurrences 8, 14, 15 and 38, additional file 3), which are three copies of IS*Lxc*2 and one copy of IS*Lxc*3, have only one of the adjacent sequences homologous to the *Lxx *genome. The other end is specific to *Lxc *genome, indicating an association with genomic rearrangements. Although some IS elements of both genomes belong to the same families and may recognize the same target site for insertion, none of the *Lxc*-IS insertion loci were the same as for elements found in the *Lxx *genome (Fig. 2 and Additional file 3). The 12 remaining *Lxc*-elements were inserted in specific regions of its chromosome (Additional file 4).

IS elements were randomly distributed throughout *Lxx *genome (Fig. 2), 25% of all insertions were located in what has been described as genomic islands [2,23], in particular within genomic island *Lxx*GI3. Comparatively the distribution of the elements in *Lxc *also seems to be random; however our approach so far does not allow us to make the same inferences about islands in *Lxc *genome.

**Figure 3:**
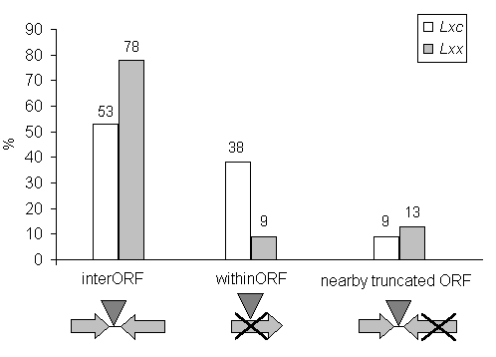
**Classification of IS insertions according to the effect on proximal genes in *Lxc *and *Lxx***: inter ORF (intergene); within ORF; and nearby truncated ORFs. The orientation of adjacent genes or genes invaded by IS elements were not considered. Percent-values are relative to all IS elements present in *Lxx *genome and in our *Lxc *dataset.

To assess the impact of IS elements in gene disruption, they were classified into three categories: IS insertions within predicted genes; insertions in non-coding sequences; and insertions in non-coding sequences, but with one or two truncated genes nearby (less than 100 nucleotides distant) (Fig. 3). Truncated genes were defined as those ORFs that have a disrupted coding sequence based on BlastX results. Most of the elements were found inserted within intergenic regions for both genomes. In the *Lxx *genome, disrupted genes were associated mainly with degradation of polysaccharides, transport and regulatory functions, while in the *Lxc *genome they were linked to cell structure, regulatory functions and hypothetical genes (Additional file 3 and 4). Five of the putative genes truncated in *Lxc *have truncated orthologs in the *Lxx *genome however, in *Lxx *no IS insertions were detected: three of them probably encoded hypothetical proteins, Lxx05470 (*Lxx *genome position: 552,768–555,523), Lxx17040 (1,767,422–1,768,564) and Lxx21675 (2,229,230–2,230,675); another one, Lxx01850 (180,918–182,055), presented a conserved acyltransferase domain, associated with lipopolysaccharide modification; and the last one, Lxx14740 (1,531,157–1,531,354), presented a DNA-binding domain. Within *Lxc*, those genes were invaded by IS*1237*, IS*Lxc*2, IS*Lxc*3, IS*1237 *and IS*Lxc*4, respectively. No DRs were detected within *Lxx *orthologous that could be an indicative of a prior invasion and excision event. Therefore is more likely that these genes were truncated prior to the IS elements insertion in *Lxc*.

### Distribution of *L. xyli*-IS-related elements throughout Actinobacteria

**Figure 4:**
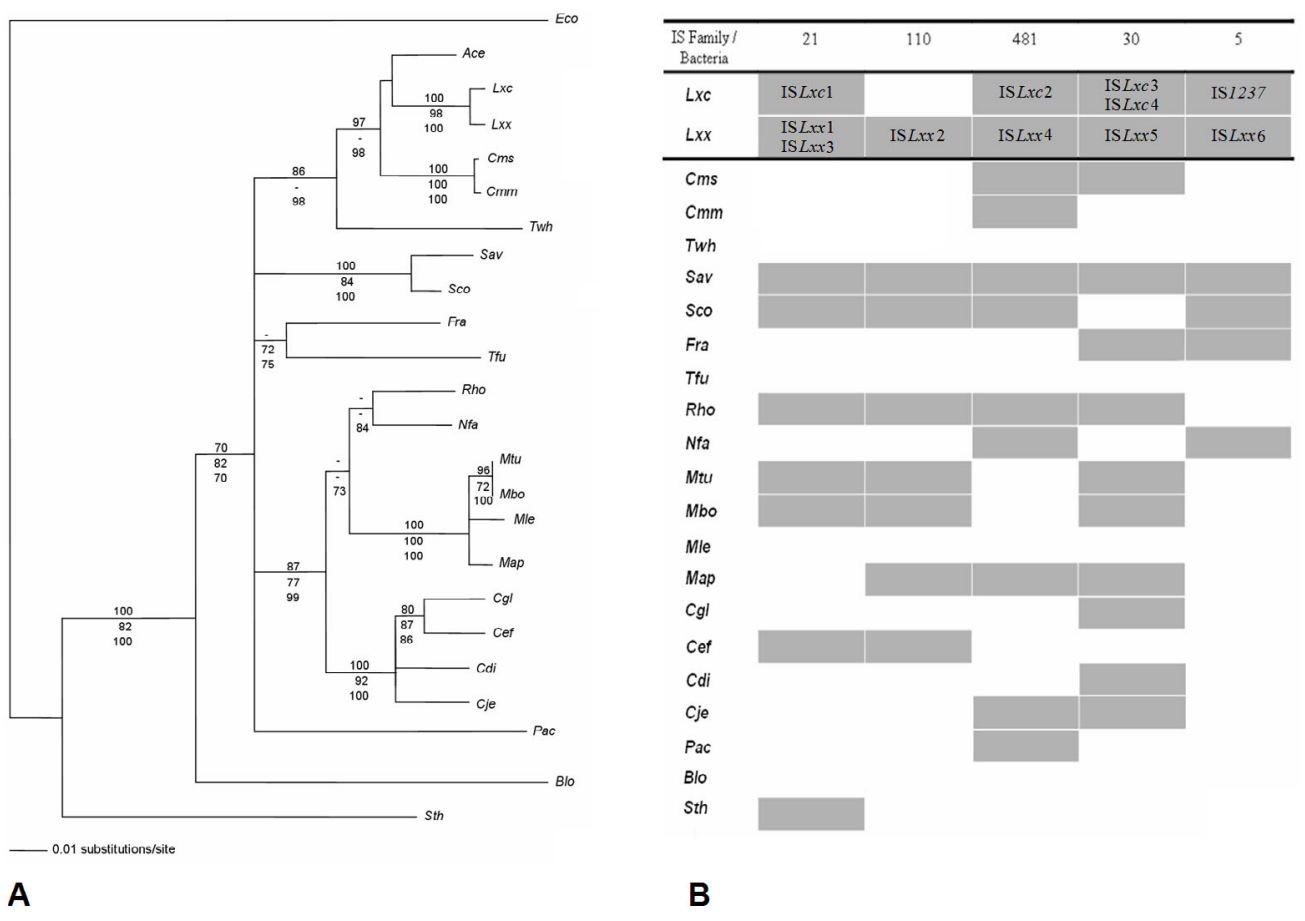
**Comparative analysis of *Leifsonia xyli *IS elements with putative transposases of Actinobacteria**. (A) Actinobacteria were arranged based on their phylogenetic relatedness. Consensus neighbor-joining phylogenetic tree was constructed using SSU rDNA(16S) sequences of: twenty-two completely sequenced genomes; in addition to *Agromyces *sp., which was reported as forming a coherent cluster to *Leifsonia *[44,45]; and *E. coli *as a outgroup. Numbers at the branches represent bootstrap values for parsimony (top, 2000 replicates), maximum likelihood (middle, 100 replicates) and neighbor-joining (bottom, 2000 replicates). Only bootstrap values above 70 are shown. (B) IS elements characterized from *Lxc *and *Lxx *genomes were used as *in silico *probes for the identification of distribution of IS families belonging to *Leifsonia xyli *genomes in other Actinobacteria. The ISfinder database was also searched for the presence of IS families in the Actinobacteria assayed here. Species names and accession numbers are listed: *Eco*-Escherichia coli K12 (NC_000913); Ace – Agromyces sp. (AM410680); Lxc – Leifsonia xyli subsp. cynodontis DSMZ46306 (unpublished); Lxx – Leifsonia xyli subsp. xyli CTCB07 (NC_006087); Cms – Clavibacter michiganensis subsp. sepedonicus ATCC33113 (NC_010407); Cmm – Clavibacter michiganensis subsp. michiganensis NCPPB 382 (NC_009480); Twh – Tropheryma whipplei str. Twist (NC_004572) and TW08/27 (NC_004551); Sav – Streptomyces avermitilis MA-4680 (NC_003155); Sco – Streptomyces coelicolor A3(2) (NC_003888); Fra – Frankia sp. CcI3 (NC_007777); Tfu – Thermobifida fusca YX (NC_007333); Rho – Rhodococcus sp. RHA1 (NC_008268); Nfa – Nocardia farcinica IFM 10152 (NC_006361); Mtu – Mycobacterium tuberculosis H37Rv (NC_000962) and CDC1551 (NC_002755); Mbo – Mycobacterium bovis AF2122/97 (NC_002945); Mle – Mycobacterium leprae TN (NC_002677); Map – Mycobacterium avium subsp. paratuberculosis str. k10 (NC_002944); Cgl – Corynebacterium glutamicum ATCC 13032 (NC_006958); *Cef *– *Corynebacterium efficiens *YS-314 (NC_004369); *Cdi *– Corynebacterium diphtheriae NCTC 13129 (NC_002935); Cje – Corynebacterium jeikeium K411 (NC_007164); *Pac *– *Propionibacterium acnes *KPA171202 (NC_006085); *Blo *– Bifidobacterium longum NCC2705 (NC_004307); Sth – Symbiobacterium thermophilum IAM 14863 (NC_006177).

According to the ISfinder database, elements of the IS*5 *family are common in both Archaea and Eubacteria [12]. Considering all IS families found in *Leifsonia xyli*, examining ISfinder submission reports and using sequence similarity searches, we found that IS5 elements were the least represented in the sequenced genomes of Actinobacteria, despite being the most highly represented IS element in the *Lxc *genome. As observed in *L. xyli *genomes, transposases from the IS*30 *and IS*481 *families are abundant in most Actinobacteria (Fig. 4). Each putative transposase of characterized IS elements of *Leifsonia xyli *genomes was used as query and compared to transposases of Actinobacteria genomes, and the best BlastX hit result was considered. Most of the transposases shared around 70% of amino acid identity. The exception was IS*1237*, which related elements of Actinobacteria genomes did not share more than 50% identical amino acids. Actinobacteria is one of the largest taxonomic units in terms of number and variety of species [34] and members of IS elements of most characterized families are represented within this group [12]. As probably expected no correlation was found between phylogenetic relatedness and IS family distribution in Actinobacteria genomes (Fig. 4). Not even among subspecies of *Leifsonia xyli *and *Clavibacter michiganensis*, which are much related to each other. This observation is in concert with the cycles of expansion and extinction of IS elements proposed before [14].

### Distribution of IS elements in *Lxc *isolates

**Figure 5:**
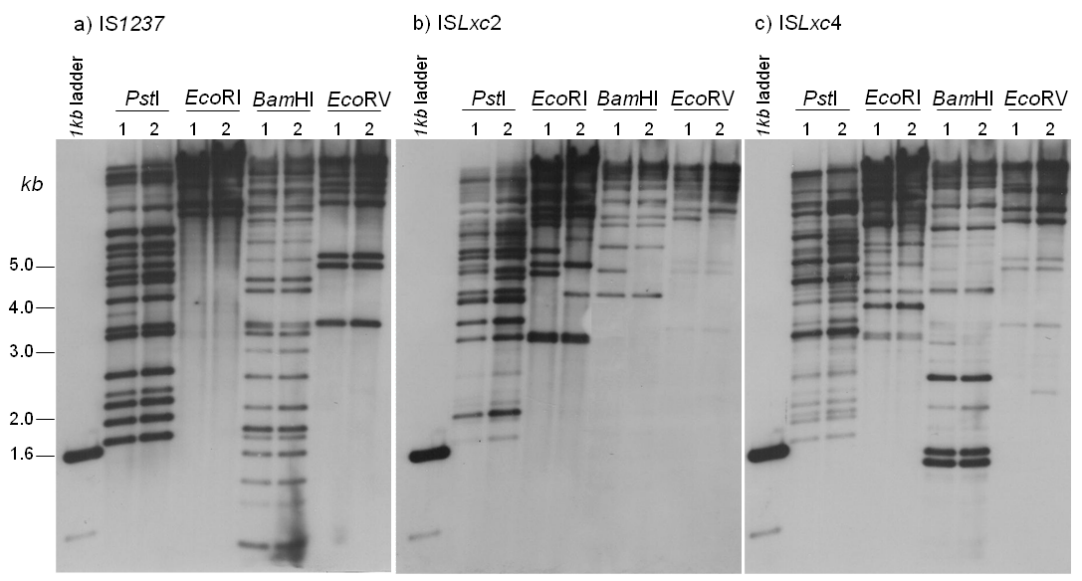
**Southern hybridization of digested DNA of two *Lxc *isolates**. The isolates used were (1) DSMZ46306 and (2) SB with probes (a) IS*1237*, (b) IS*Lxc*2 and (c) IS*Lxc*4. The DNA molecular marker used was a *1kb *ladder (Invitrogen, Carlsbad, CA).

To further examine the distribution of IS elements between genomic DNA of two isolates of *Lxc *to check the involvement of these elements in diversification of strains, hybridization experiments were performed using fragments of three IS elements of different families as probes. There are two *Bam*HI sites within IS*1237*, one within IS*Lxc*4, and none within IS*Lxc*2. There are no sites for *Pst*I, *Eco*RV or *Eco*RI within these elements. IS*1237 *is the only element showing the same hybridization pattern between the two isolates for all tested enzymes (Fig. 5). However, variable DNA band patterns were detected for IS*Lxc*2 and IS*Lxc*4 (Fig. 5). We believe that partial digestions can not justify such differences because the same stripped membrane was used in each experiment, and we do not see partials in the assay with IS*1237*. Also, polymorphism at the enzymes restriction site could not explain the differences since they were found for all enzymes used when probed with IS*Lxc*2 and IS*Lxc*4, and they were not detected for IS*1237*, mainly considering the abundance of the later in *Lxc *genome. Finally, we assume that these differences were probably not the result of large-scale rearrangements; otherwise they would also have been detected in the distribution of IS*1237*. It is plausible to assume that the observed variation may result from homologous recombination between copies of these elements not interspersed by *IS1237 *copies, or that it may be associated with the presence of a variable number of elements, as a result of transposition of IS*Lxc*2 and IS*Lxc*4 to new locations. To assure any of these two hypotheses the bands should be isolated and the DNA sequenced. In any case, the difference exists and is probably originating diversification in the isolates of *Lxc *genomes. Consistent with this are the data generated by Young and collaborators where isolates of *Lxc *showed different band pattern in DNA fingerprinting [35].

## Discussion

Sequencing of approximately 50% of the *Lxc *genome identified 56 copies of five distinct IS elements, establishing that the *Lxc *chromosome contains a larger number of IS copies than the *Lxx *genome [2]. The IS elements of *Lxc *were subjected to a careful analysis and compared to the elements of *Lxx *and other Actinobacterial genomes, in an attempt to understand their contribution to the genome diversification of the two *L. xyli *subspecies. Also, taking into account that genome projects usually fail in a detailed characterization of these elements, we reviewed all the elements in both *Lxc *and *Lxx *genomes.

Apart from sharing the same IS families, there were no iso-forms in common between the *Lxc *and *Lxx *and the locations of insertions were distinct to each genome. Analysis of the impact of IS insertions revealed that, in both subspecies, most elements were inserted within intergenic regions. The higher percentage of this kind of insertion in *Lxx *(78%) as compared to the *Lxc *genome (53%) is probably due to the *Lxx*-genome decay process, since a higher percentage of genes were already non-functional, as previously proposed [2]. Insertions within genes or intergenic regions were independent of IS family assignment for both subspecies. The *Lxc *and *Lxx *genome comparative arrangement was assessed by mapping the flanking sequences of each *Lxc*-IS element in the complete *Lxx *sequence; some elements were associated with DNA rearrangements and others were inserted into specific fragments of the *Lxc *genome.

IS*1237 *is an unique element, with some similarity to elements of *Streptomyces coelicolor *A3(2) (NP_624431), *S. avermitilis *MA-4680 (NP_821293) and *Frankia *sp. CcI3 (YP_481128). An expansion of it was detected in the *Lxc *chromosome, whereas elements of the IS*5 *family were the least represented in Actinobacteria. Particularly intriguing is the presence of a single copy of an IS*1237*-related element within *Lxx *genome, the IS*Lxx*6. Despite sharing 85% of nucleotide identity with IS*1237*, the transposase of IS*Lxx*6 appears to be intact. One would probably expect a larger expansion of it within *Lxx *genome, but somehow its transposition may be controlled by mechanisms other than that regulated at translational level. This is probably an interesting model to be further analyzed. IS elements are very often plasmid-borne, and transfer events between genomes and plasmids are common [12], which may be the case of IS*1237*, first described in the cryptic plasmid pCXC100 present in some of the *Lxc *isolates [24]. The extended survey of all *Lxc *and *Lxx*-IS elements among Actinobacteria genomes showed no correlation between phylogenetic relatedness and distribution of IS families, which is probably in agreement with the hypothesis that genomes undergo repetitive extinction-reinfection cycles of different IS elements throughout bacterial evolution [14].

Most of the IS families assigned in *L. xyli *were characterized by maintaining transposition at low levels using mechanisms, such as regulation at the translational level [21], *i.e*., transposition occurs only when translation overcomes features of the sequences such as sites for ribosomal slippage and premature termination codons. So far, copies of *Lxc *and *Lxx *elements sequenced within each genome have proved almost identical, therefore, we have assumed that these features are part of the mechanism to regulate transposition, rather than being indicative of defective IS elements [21].

To assess the involvement of *L. xyli *IS elements carrying these types of transposases in promoting genome reorganization, the distribution of three IS elements of different families was analyzed in two strains of *Lxc*. The observed band polymorphism raised the hypothesis that IS*Lxc*2 and IS*Lxc*4 are involved in diversification, either by being active in transposition or by promoting homologous recombination. Variation was not observed for IS*1237 *hybridization, supporting previous experimental data where mobilization *in vitro *was not achieved [25]. Conversely, the elevated number of identical copies within the *Lxc *chromosome, the identical positioning in different isolates, and insertions within other elements suggest recent expansion of *IS1237*.

Transposon activity is regarded as an important source of bacterial diversity not only in promoting gene inactivation and genome reorganization but also in acquisition of new sequences [12]. Consequently, there must be some equilibrium between the impact of those events in successful maintenance of the element and host viability. How the differential expansion happens even among close related species and strains remains to be fully understood.

## Conclusion

The impact of IS elements on genomic organization and gene content among closely related species has previously been described [16,17], and our data further support such analyses. Although the approach used to sequence the genome of *Lxc *was limited, an extensive study using the data gathered was sufficient to show that the genomic diversification of *Lxc *and *Lxx *is also and perhaps primarily associated with the presence of distinct types of IS elements. In addition to that, we have made a detailed characterization of IS elements present in each genome, which is often missing in analysis of fully sequenced genomes. The set of IS elements being unique to each genome, their specific location in combination with rearrangements and horizontal gene transfer are probably the major forces of genome evolution in *Leifsonia xyli *and consequently should have an impact on its biology. The same is probably true for isolates of *Lxc *as determined by our hybridization experiments. Our study also provided information in concert with the concept that distribution of IS elements in a given genome happens in evolutionary recent events due to cycles of expansion and extinction.

These data will probably contribute to our ongoing comparative analyses aimed at understanding the biological differences of the *Lxc *and *Lxx *genomes.

## Methods

### Bacterial strains and growth conditions

The bacterial strains used were: *Lxx *CTCB07 (NC_006087) [2] (Brazil); *Lxc *DSMZ46306 (Taiwan); and *Lxc *SB (Australia). The DSMZ46306 strain was grown in a *MSC New *modified liquid medium [3,2] at 28°C for 5–10 days, under agitation at 300 rpm. The SB genomic DNA was kindly provided by Dr. Steven Brumbley (Bureau of Sugar Experiment Station/Queensland, Australia).

### Genomic library construction, DNA sequencing and assembly

Two DSMZ46306 genomic libraries were prepared. Genomic DNA extracted as previously described [2] was mechanically sheared and the resulting fragments cloned into pUC19 (Q-Biogene, Carlsbad, CA). A BAC clone library was prepared with 25 kbp-inserts obtained by partial digestion with *Bam*HI [36] cloned into *pIndigoBAC-5 *(Epicentre^®^). Both ends of each insert were sequenced using an automated sequencer (model 3700, ABI Prism, Applied Biosystems, Foster City, CA). Results were analyzed by ABI sequencing analysis software, and assembled using the *phred/Phrap/consed *package [37,38]. All consensus sequences were generated with *phred *quality ≥ 20.

### Characterization of IS elements

Inserts containing end-sequences similar to transposases were subcloned or primer-walked. Homology searches were performed using BlastN and BlastX [39] at GenBank [40] and IS finder [41]. Identities were considered significant only when the E-value was less than 10^-05^. Positive matches for transposase/integrase were manually verified to determine the presence of the following determinants of a given family: element size; presence of terminal inverted repeats (IR) and conserved terminal based pairs; target site of insertion and number of bases associated to direct repeats (DR); number of ORFs; distance among amino acids of DDE; as well as comparisons with related elements [11]. We also analyzed the presence of variants, domains, frameshifts and premature stop codons.

Transposases sharing more than 95% of amino acid identity were grouped. Their nucleotide sequences in addition to 300 bases up and downstream were aligned using ClustalW [42]. For each cluster of sequences, the first sixty bases of the 5'-end was aligned with the reverse complement of the first sixty bases of the 3'-end. Best matches were considered as IR candidates and compared to IRs of other characterized IS elements. IRs were selected when the minimum number of non complemented nucleotides followed the pattern of a given family. In an attempt to identify the target sites of insertion and the DR, an alignment of up to 20 bases flanking each IS element was done and the sequence was compared to the already described target sites of other IS elements. Features such as stop codon in frame and purine rich sites related to ribosome frameshift were sought in ORFs whose alignment partially matches transposases, based on BlastX results. Domains were identified within the Blast results at the CDD database [40]. DDE motifs were identified following IS finder pattern [41]. Minimal numbers of occurrences were determined based on flanking sequences.

### Comparing *Lxx *and *Lxc *sequences

*Lxc*-IS element loci were compared to those of the *Lxx *genome using adjacent sequences as anchors and a cross_match program, which is part of the swat/cross_match/phrap package [38], with default parameters. *Lxc*-specific loci were annotated using SABIA [43].

### Analysis of IS element distribution within Actinobacteria

Amino acid sequences of putative transposases were used as queries within completely sequenced genomes of Actinobacteria. Genomes were downloaded from GenBank (IDs in Fig. 4). Searches were performed using BlastX and tBlastN [39].

### Southern blot hybridization

*Lxc *genomic DNA (1 μg; DSMZ46306 and SB) was digested completely with *Pst*I, *Eco*RI, *Bam*HI and *Eco*RV. Fragments were separated in a 0.8% (w/v) agarose gel and transferred to a Hybond N+ membrane (Amersham, Piscataway, NJ). Probe labeling, hybridization and detection were performed with an ECL kit (Amersham) according to the manufacturer's guidelines and under high stringency conditions. Inserts of shotgun clones containing the entire fragments of three *Lxc*-IS elements (IS*1237*, IS*Lxc*2 and IS*Lxc*4) were amplified and used as probes. The same membrane was stripped and re-used according to the manufacturer's recommendations.

## Authors' contributions

MMZ: carried out the molecular genetic studies, prepared the *in silico *analysis and drafted the manuscript. M–AVS: Data interpretation and analysis and helped to draft the manuscript. LEAC: Data interpretation and analysis and helped to draft the manuscript. CBM–V: conceived of the study, and participated in its design and coordination and helped to draft the manuscript. All authors read and approved the final manuscript.

## Supplementary Material

Additional file 1 Comparative analysis of *Lxc*-IS elements and the most related IS element of *Lxx *genome. Click here for file

Additional file 2 Characterization of IS elements of *Leifsonia xyli *subsp. *cynodontis*. Click here for file

Additional file 3 Mapping of *Lxx *(gray lines) and *Lxc *(white lines) IS elements within the *Lxx *genome based on their flanking sequences. Click here for file

Additional file 4 IS elements inserted in specific fragments of *Lxc *genome. Click here for file

Additional file 5 Details of IS*Lxx*6 and IS*1237 *sequence alignment and putative transposase amino acid sequences. Click here for file
