# Supplementary material for: Characterization of new IS elements and studies of their dispersion in two subspecies of Leifsonia xyli
Source: BMC Microbiol. 2008 Jul 25;8:127. doi: 10.1186/1471-2180-8-127 (PMC2516522; doi:10.1186/1471-2180-8-127)
Supplement: Additional file 1 — Comparative analysis of Lxc-IS elements and the most related IS element of Lxx genome. [file 1471-2180-8-127-S1.pdf]

Additional file 1 - Comparative analysis of *Lxc*-IS elements and the most related IS element of *Lxx* genome.

Lxc-IS (GenBank ID)	Most related *Lxx*-IS	Percentage of identity/similarity with *Lxx* IS elements	
		Nucleotides[a] (whole element)	Amino acids[b] (ORF only)
IS1237 (X75973)	IS*Lxx*6	85%	78%
IS*Lxc*1 (DQ191803)	IS*Lxx*3	71%	75%
IS*Lxc*2 (EF176596)	IS*Lxx*4	70%	63%
IS*Lxc*3 (EF421582)	IS*Lxx*5	71%	69%
IS*Lxc*4 (EF433175)	IS*Lxx*5	57%	52%

[a] Nucleotides sequences were aligned across their entire length, from IR to IR (global alignment).
[b] Amino acid sequences were aligned using Blast2seq (local alignment) (http://www.ncbi.nlm.nih.gov/blast/bl2seq/wblast2.cgi)
