# Supplementary material for: Characterization of new IS elements and studies of their dispersion in two subspecies of Leifsonia xyli
Source: BMC Microbiol. 2008 Jul 25;8:127. doi: 10.1186/1471-2180-8-127 (PMC2516522; doi:10.1186/1471-2180-8-127)
Supplement: Additional file 2 — Characterization of IS elements of Leifsonia xyli subsp. cynodontis. [file 1471-2180-8-127-S2.pdf]

Additional file 2 - Characterization of IS elements of *Leifsonia xyli* subsp. *cynodontis*.

IS*1237*

```
(CGCGGCGGAACCGCGATTTA)
GAGGTGGTTTCAGTAGTCGGCGTGGCGGTGGTTGTTGTGGCTGGTTAGCGGGGATGCTGGTCGAGTGTCG
ACGCTTGCTGAGGATCGGTTCATTACGGATATGTTGTGGGAGCGGCTGGAGCCGTTGATTCCGCCTCGGC
CGCCTGTGGTCAATGGGCGGGCTGGGCAGCCTCGGGTTCCTGACCGGAAGGTGTTCGCTGGGATCGTGTT
CGTGCTGCTGACGGGGATCCC GTG GAG AAG CTC CCG CCC GAG TTG GGG TAT GGG TCC
                       M   E   K   L   P   P   E   L   G   Y   G   S
 GGG GTC ACT TGT TGG CGG CGT CTG CGT GAA TGG TCC GAA GCG GGC GCG TGG
  G   V   T   C   W   R   R   L   R   E   W   S   E   A   G   A   W
 GAT GCA CTG CGG AAG ATC ATG CTC GAC GAA CTC GGC CAG GCT GGC ATG ATC
  D   A   L   R   K   I   M   L   D   E   L   G   Q   A   G   M   I
 GAC TGG TCA AGA ACC TGC CTG GAC TCC GTA AGT GTC CGG GCG AAA AGG GG
  D   W   S   R   T   C   L   D   S   V   S   V   R   A   K   R   G
 GGC GAT CTC ACT GGA CCT AAC CCC ACG GAT CGT GGG AAA CGG GGC ACC AAG
  G   D   L   T   G   P   N   P   T   D   R   G   K   R   G   T   K
 TAC CAT GTC CTG ACC GAC CGC AAC GGA CTC CCG CTG CAT GTG GAG ATC TCC
  Y   H   V   L   T   D   R   N   G   L   P   L   H   V   E   I   S
 GGC GCC AAC CGA CAC GAC TCC ATG CTC GTG GAA CCT GTG TTA GAC AAC ATC
  G   A   N   R   H   D   S   M   L   V   E   P   V   L   D   N   I
 ACC GCG ATC AAG GGC GTC GGC CGC GGT CGG CCC AGA CGC CGC CCG GTT ATC
  T   A   I   K   G   V   G   R   G   R   P   R   R   R   P   V   I
 TTC CAC GCC GAT AAG GCT TAC GAC AAC CGC CGC GTC CGC TGT TAC CTG CGT
  F   H   A   D   K   A   Y   D   N   R   R   V   R   C   Y   L   R
 TGT CGT GGG ATC AAG GCA CGC ATC GCA CGA ATC GGA GTC GAC TCC AAA CAG
  C   R   G   I   K   A   R   I   A   R   I   G   V   D   S   K   Q
 TGA CTG GGT AAA CAC TGT TGG GTA GTC GAA CGC ACC ATG GCC TGG ATC CTC
  *   L   G   K   H   C   W   V   V   E   R   T   M   A   W   I   L
 GCC TTC CGG AAA CTC GCC ACT CGC TAC GAC CGC ACC GCC TCA ACG ATC ACG
  A   F   R   K   L   A   T   R   Y   D   R   T   A   S   T   I   T
 GCG CTC GTC GCT CTA GCA ATC GCG ATC ACC AGC GCC CGC AAA CTC ACC AAA
  A   L   V   A   L   A   I   A   I   T   S   A   R   K   L   T   K
 AAC GAC TAC TGA AACCATCTC
  N   D   Y
(TTATAGCAGAGCACGAAGAG)
```

Sequence 1. IS*1237* (899 bp version). (a) In between parenthesis: adjacent sequences of one copy (4º occurrence – additional file 3); **(b)** letters highlighted in gray: DR; **(c)** letters highlighted in green and non complementary nucleotides highlighted in yellow: IR; **(d)** letters in red: transposase start and end; **(e)** letters beneath nucleotide sequence separated in triads: transposase coding sequence; **(f)** letters highlighted in brown: purine rich region possible associated with -1 frameshifting; **(g)** letters in boldface: missing sequence of IS1237d1 (798 bp); **(h)** letters in pink: premature stop codon; **(i)** letters within rectangles: DDE motif and **c**onserved amino acids of IS5 family.

IS*Lxc*1+IS*1237*

(AGCAGCAGCTGGCGATCGCC)
TGTCTGTGTCACTGTTAGCGGGCTCCGGGTTGTCGGAGTTGTCTGGCCCCGGCGGCGACTTGCCGGGCTG
GTTGTGTCGAAGCTAA**GAGGTGGTTTCAGTAGTCG**GCGTGGCGGTGGTTGTTGTGGCTGGTTAGCGGGGA
TGCTGGTCGAGTGTCGACGCTTGCTGAGGATCGGTTCATTACGGATATGTTGTGGGAGCGGCTGGAGCCG
TTGATTCCGCCTCGGCCGCCTGTGGTCAATGGGCGGGCTGGGCAGCCTCGGGTTCCTGACCGGAAGGTGT
TCGCTGGGATCGTGTTCGTGCTGCTGACGGGGATCCCGTGGAGAAGCTCCCGCCCGAGTTGGGGTATGGG
TCCGGGGTCACTTGTTGGCGGCGTCTGCGTGAATGGTCCGAAGCGGGCGCGTGGGATGCACTGCGGAAGA
TCATGCTCGACGAACTCGGCCAGGCTGGCATGATCGACTGGTCAAGAACCTGCCTGGACTCCGTAAGTGT
CCGGGCGAAAAGGGGGGCGATCTCACTGGACCTAACCCCACGGATCGTGGGAAACGGGGCACCAAGTACC
ATGTCCTGACCGACCGCAACGGACTCCCGCTGCATGTGGAGATCTCCGGCGCCAACCGACACGACTCCAT
GCTCGTGGAACCTGTGTTAGACAACATCACCGCGATCAAGGGCGTCGGCCGCGGTCGGCCCAGACGCCGC
CCGGTTATCTTCCACGCCGATAAGGCTTACGACAACCGCCGCGTCCGCTGTTACCTGCGTTGTCGTGGGA
TCAAGGCACGCATCGCACGAATCGGAGTCGACTCCAAACAGTGACTGGGTAAACACTGTTGGGTAGTCGA
ACGCACCATGGCCTGGATCCTCGCCTTCCGGAAACTCGCCACTCGCTACGACCGCACCGCCTCAACGATC
ACGGCGCTCGTCGCTCTAGCAATCGCGATCACCAGCGCCCGCAAACTCACCAAAAA**CGACTACTGAAACC**
ATCTCTAAGTGGCCCCACCTGAGTGTGGTTCCCGTCTGTTACTTCAGGATGGGAGGGCCCGGGTTGGGGT
CGAGGTTGGATCAGTTCGCTGCGATCAGGAGAGATGCCCGGGTGGAGGGTCTCTCTATTCGTGAGCTTGC
TGTTCGTCATGGTGTTCACCGGCGGACGGTTCGTCAGGCTTTGGAGTCAGCGACGCCACCGTCGCGGAAG
CCGAGAGTTCAGTCGTCGCCGAAGCTGGATCTGGTTCGCGAGCTGATCGACGCGATTCTGCGTCAGGATC
TCGGGGCGCCGAAGAAGCAGAAGCAGACGGCGACACGGATCTGGAAACGTCTCCTCGATGAGCACCAGGT
CGATGTCTCCTACGGCACGGTCCGTGACTATGTGCGCTCCCGGCGCCCGCAGATCGACGCGGAGGCTGGT
CGTCTGCCGGAGGTGTTCGTCCCGCAGGAGCATGCTCCGGGGGCGGAGGCGGAGGTCGATTTCGGTGAGG
TCTGGGTGATCCTGGCCGGGGTGAAGACCAAGTGCCACATGTTCACTTTTCGGCTCTCGTACTCGGGGAA
GGCGATTCACCGGGTCTACTCGACGCAGTCGCAAGAGGCGTTCCTGGAAGGCCACATCGACGCGTTCGAG
GAGATTGGCGGGATGCCGACCCTTCACATCAAATACGACAACTTGGCCGCCGCGGTGAAGTCCGTGGTCA
ACGGCAAGGACCGCAAACGGGTCGAGAATGACCGTTGGGTCCTGTTCCGTTCCCACTACGGATTCGACGC
GTTCTACTGCCAGCCCGGCATTGACGGTGCGCACGAGAAGGGCGGCGTTGAGGGCGAGGTCGGCCGGTTC
CGCCGCACGTGGCTCTCACCCATGCCCGAAGTCGACTCCCTCGCGCAACTGAACGCGATGATCCGCCGCT
GGGATGCCCGTGACGAGCAGCGCCGGATCGCGCAGCGCCGAACCAAGGTCGAGGACGACTTCGCTACAGA
ACGCCCGCTCCTTCGGCCGCTGCCGGCGGAGCGATTTGATCCGGGACTGGTGCTGCATCCTCGGGTCGAC
CGGTCCGGGCTGATCACGGTCCGGATGGCGAAATACTCTGTCCCTGCCCGGCTCATCGGCCGTGAAGTCC
GCGTCTCTCCGCGCTTCCGAGGTCGTCGTCTTCGACGGGCGCGTTGAAGTCGCCCGGCACGAACGCGTGG
TCGCCCGCGGAGGCGAGTCGATCCAGCTGGATCACTACCTCGAGGTCCTCCGGCACAAGCCCGGCGCGTT
CCCCGGCTCGACAGCACTTGCACGCGCCCGGGAGGCGGGAACCTTCACCGCCGCCCATGAAGCGTTCTGG
CAGGAGGCAAGGAAAGTCAATGGCGACACGGCCGGCACCAGAGAGCTCGTCGACGTGCTCCTGCTCCATC
GCAGCATGCGCGCAGCCGACGTGATCGCCGGGATCCGCGCGGCACTCTCGGTCGGGGCCATCTCCGCTGA
CGTCGTCGCGGTTGAAGCACGACTGTACGCAGGTGGGGCCATCCAACACCGACAGCCCGTTGAACAACGC
CCCGATCGCGAGCGACGAGTTGTCAGTCTCACCCAGCGCCGGCTCCGGGATCCGCAGGCTGTCATCGCCG
GCCTGCCACAGGACAAACGCCCGCTCCCGACCGTCACCCAATACGACGAACTCCTCCAGCGCCGCCCCGT
CTTCACCGACCCCACCACCGATTGAGAGAGAAGGAACCACCGACAC ATG AGC CCA ACC ACC ACG

```
                                              ATG AGC CCA ACC ACC ACG
                                               M   S   P   T   T   T
AAC ATC ACC ACC ACC CTC CGC CGG CAA CGC GGG ATG ACC CAG GAA GCC GCC
 N   I   T   T   T   L   R   R   Q   R   G   M   T   Q   E   A   A
GCG GCC GCC GTC GAC CAA GCC TGC AGA CGC CTG CGA CTA CCG ACC ATT CGC
 A   A   A   V   D   Q   A   C   R   R   L   R   L   P   T   I   R
GCC GTG ATG GAC GAA GCG ATC CGG GTC GCC GAG CAC GAG CAG CTG TCC TAC
 A   V   M   D   E   A   I   R   V   A   E   H   E   Q   L   S   Y
CAA GGC TTC CTC GCC GAA GTG CTG TTG GCC GAG TGC GAC GAC CGC GAC CGC
 Q   G   F   L   A   E   V   L   L   A   E   C   D   D   R   D   R
CGC TCC ACC GTC CGC CGC GTC GCC TCC GCC GGC TTC CCA CGT CAG AAA TGG
 R   S   T   V   R   R   V   A   S   A   G   F   P   R   Q   K   W
CTC GGC GAC TTC GAC TTC GAC ACC AAC CCG AAC ATC AAC GCG GCG ACC ATC
 L   G   D   F   D   F   D   T   N   P   N   I   N   A   A   T   I
CAC ACG CTC GCC ACC GGC GAC TGG GTC AGA CGC GGC GAC CCG CTC TGC CTC
 H   T   L   A   T   G   D   W   V   R   R   G   D   P   L   C   L
ATC GGG GAC TCC GGC ACC GGC AAG AGC CAC CTC CTC ATC GGC CTC GGC ACC
 I   G   D   S   G   T   G   K   S   H   L   L   I   G   L   G   T
GCC GCA GCC GAG AAG GGC TAC CGA GTC AAA TAC ACC CTC GCG ACC AAG CTC
 A   A   A   E   K   G   Y   R   V   K   Y   T   L   A   T   K   L
```

```
GTG AAC GAA CTC GTC GAA GCA GCA GAT GAG AAG CAG TTG GCC CGC ACG ATC
 V   N   E   L   V   E   A   A   D   E   K   Q   L   A   R   T   I
GCT CGC TAC GGC CGC GTC GAT CTG CTC TGC ATC GAC GAG CTC GGC TAC ATG
 A   R   Y   G   R   V   D   L   L   C   I   D   E   L   G   Y   M
GAA CTC GAC CGA CGC GGC GCC GAG CTC CTC TTC CAA GTC CTC ACC GAA CGC
 E   L   D   R   R   G   A   E   L   L   F   Q   V   L   T   E   R
GAA GAG AAG AAC TCC GTC GCG ATC GCA TCC AAC CAG TCA TTC TCG GGA TGG
 E   E   K   N   S   V   A   I   A   S   N   Q   S   F   S   G   W
ACG GAC ACC TTC ACC GAC CCC AGG CTC TGC GCT GCC ATC ATC GAG ACC GGC
 T   D   T   F   T   D   P   R   L   C   A   A   I   I   E   T   G
ACC ACC TCC TAC CGC CTC CAA CAC ACC CGC AAC ACC GCA CTC GCT GGG GCC
 T   T   S   Y   R   L   Q   H   T   R   N   T   A   L   A   G   A
AAC TAA CTTCGACAACCCGGTCCCCGCCAGCCTTGACATAGTCA
 N
(ATCGCCCGGCTCCGCGCCAT)
```

Sequence 2. IS*Lxc*1 (2,631 bp) and the IS*1237* insertion represented in white letters highlighted in black. **(a)** In between parenthesis: adjacent sequences of one copy (11º occurrence – additional file 3); **(b)** letters highlighted in gray: DR; **(c)** letters highlighted in green and non complementary nucleotides highlighted in yellow: IR; **(d)** letters in pink: premature stop codon of the disrupted *ist*A; **(e)** letters in red: *ist*B start and end; **(f)** letters beneath nucleotide sequence separated in triads: IstB coding sequence; (**g**) letters highlighted in brown: purine rich region.

IS*Lxc*2

```
(CTCGTCGCCGGGGTCTAGGT)
TGTGCTGCTCAGGGACGTTGGTTGAGGTGTGACGCGATAGATGGGTGAGGACCTCCCGGTCGAGAGTGGG
GCTGTCTAGTTTCCCTGCACTCGATGACTTAGGAGGTCCTC GTG ACC CAC GCT AAT GCT GCT
                                           M   T   H   A   N   A   A
TTG ACT CCT CGC GAA TGC CTC CGC CTG GCC CGC CAA GTC GTC GAC GAC GGC
 L   T   P   R   E   C   L   R   L   A   R   Q   V   V   D   D   G
TGG TCC GTT GCT GCG GCG GCG ACC TAC TTC CGA GTG TCC TGA CGC ACC GCG
 W   S   V   A   A   A   A   T   Y   F   R   V   S   *   R   T   A
GAC CGA TGG GCT CGT CGT TAC GTG GAG ATG GGC GAG GCG GGA ATG CTG GAC
 D   R   W   A   R   R   Y   V   E   M   G   E   A   G   M   L   D
CGT TCG TCA CGG CCG CAT CAC AGC CCG AAC AAG ACC CCG CGA AGA CTG GTC
 R   S   S   R   P   H   H   S   P   N   K   T   P   R   R   L   V
CGC AAG GTC GTG CAT CTG CGG TGG AAG AAG CGG CTG GGA CCA GTC GGT ATC
 R   K   V   V   H   L   R   W   K   K   R   L   G   P   V   G   I
GGC GCC CAG CTC GGC ATG CCC GCC TCG ACC GTT CAC ACG GTC CTC TCC CGG
 G   A   Q   L   G   M   P   A   S   T   V   H   T   V   L   S   R
TGC CGG ATC AAT CGG CCC AGC CAC GTC GAC GTC CGC ACC GGC GAA CCC GCC
 C   R   I   N   R   P   S   H   V   D   V   R   T   G   E   P   A
CGC CGC TAC GAG CAC GAG CAT CCC GGA TCG ATG ATC CAC GTC GAC ATC AAG
 R   R   Y   E   H   E   H   P   G   S   M   I   H   V   D   I   K
AAA CTC GGC AAC ATC CCC GAC GGT GGC GGC TGG CGC TAC GTC GGA CGT CTC
 K   L   G   N   I   P   D   G   G   G   W   R   Y   V   G   R   L
CAG GGA GAG CGG AAC AAG GCC ATC ACC GCG AAG CGG ACC GGG AAA CAC GGG
 Q   G   E   R   N   K   A   I   T   A   K   R   T   G   K   H   G
ATC ACC GGC GAC ATG ATC ACC GGC ACA GCG TTC GTT CAT ACC GTC ATC GAC
 I   T   G   D   M   I   T   G   T   A   F   V   H   T   V   I   D
GAT CAC TCC CGT GTC GCT TAC GCC GAG ATC CAC GAC GAC GAA ACC GCC GCC
 D   H   S   R   V   A   Y   A   E   I   H   D   D   E   T   A   A
ACT GCA ATC GCT GTT CTG CGT CGA GCG GTC GGC TGG TTC GCC AGC CGT GGC
 T   A   I   A   V   L   R   R   A   V   G   W   F   A   S   R   G
GTC ACC GTC GAA CAG GTG CTC TCC GAC AAC GGC TCC GCA TAC CGC TCA TAC
 V   T   V   E   Q   V   L   S   D   N   G   S   A   Y   R   S   Y
GCC TGG CGC GAC GCT TGC GCC GAG CTC AGC ATC CAA CCG AAA CGC ACC CGG
 A   W   R   D   A   C   A   E   L   S   I   Q   P   K   R   T   R
CCC TAC CAT CCG CAG ACG AAC GGC AAG ATC GAA CGC TTC CAC CGC ACC CTC
 P   Y   H   P   Q   T   N   G   K   I   E   R   F   H   R   T   L
GCC GAC GGC TGG GCA TAC GCC CGG CAC TAC AAC TCC GAA TCA GCC CGC CGC
 A   D   G   W   A   Y   A   R   H   Y   N   S   E   S   A   R   R
AAC GCA CTC CCG GCC TGG CTG CAC TCC TAC AAT CAC CAC AGG CCC CAC ACC
 N   A   L   P   A   W   L   H   S   Y   N   H   H   R   P   H   T
GCC ATC GGC AGC CAG CCA CCC ATC AGC AGA TTG ACC AAC GTC CCT GAG AAA
 A   I   G   S   Q   P   P   I   S   R   L   T   N   V   P   E   K
CAC A(CC TAG AGCGGGACCTCCGCC)
 H    T
```

Sequence 3. IS*Lxc*2 (1,105 bp). (a) In between parenthesis: adjacent sequences of one copy (5° occurrence – additional file 3); **(b)** letters highlighted in grey: DR; **(c)** letters highlighted in green and non complementary nucleotides highlighted in yellow: IR; **(d)** letters in orange: site of insertion of IS*1237* (occurrences 17, 28 and 49); **(e)** letters in red: transposase start and end; **(f)** letters in pink: premature stop codon; **(g)** letters beneath nucleotide sequence separated in triads: transposase coding sequence; **(h)** letters within rectangles: DDE motif and other conserved amino acids of IS481 family.

IS*Lxc*3

```
(CCCGGCCAGTGCCGACGCAC)
CTTGGGTTCTAACGGTCGTTGCAACACCTGGACTTTCTGGGAGTTGTAACGACCGTGGCCCGTTTAAGGA
GCTATTCGAGGGATCGACGCTGTTTCACGCAGGCTGATAAGGACGCGTTCTTCGCGTTGTTCGAGGCGTG
TGGGAGCATCACGATCGCGGCGCGTGAGTTGGGGTTCAACCCGGCGACCTGCGGGACGTGGGTATGGAAG
GCCGGCCTGCGCAGCCAGGGGAAAACCGGGACTGGTCCGCATCCAGGAAAGGAACGCTACTTCCAGTTGC
GACGCGATGGCATCTCGCGTCGCGAAGCAGCGGCTGCCGTCGGGGTGAACATCCGCACGGCACGGGATTG
GGATAACGGTGTCCGGAAGACCGCTCATCGCCGCTATTACCCAGATGGTCGGGTCGTCGATTACAAAACA
GGT ATG ACG ACG TTT CTT GAC GGC ACC GAG ACA ATC TCG CCG GCG GCG ATC
     M   T   T   F   L   D   G   T   E   T   I   S   P   A   A   I
 TTT CAG CTG GAG AGG AAG CTC GAT CCG CGG TTC CTT TCG CTG GAG GAA CGT
  F   Q   L   E   R   K   L   D   P   R   F   L   S   L   E   E   R
 GAG CAG ATC CGG GAT CTC CTC CGC GAT GGG TTG TCA CTG CGG AGC ATC GCC
  E   Q   I   R   D   L   L   R   D   G   L   S   L   R   S   I   A
 GCC CAG CTG CGG CGG TCG CCA TCC ACG ATC AGT CGG GAA ATC TCC CGG AAC
  A   Q   L   R   R   S   P   S   T   I   S   R   E   I   S   R   N
 CGG TCC TCG ACC GGG ATC TAT CAC CCC TTC GCA GCA CAT CGC TAC TCG GCG
  R   S   S   T   G   I   Y   H   P   F   A   A   H   R   Y   S   A
 AAG CGT CGC CCA CGG CCA AAG CCG CGA AGA CTG GTG ACA GAG CTG CGG TTG
  K   R   R   P   R   P   K   P   R   R   L   V   T   E   L   R   L
 CGT GCT TTC GTT GAG TCG AAG CTG GCG TTG CGG TGG TCT CCC GAG CAG ATC
  R   A   F   V   E   S   K   L   A   L   R   W   S   P   E   Q   I
 ACT CGA GCC CTG ATC CGT CAG TTC CCG GAC GAC GTT GGG ATG CGA GTG GCG
  T   R   A   L   I   R   Q   F   P   D   D   V   G   M   R   V   A
 ACC GAG ACG ATC TAC CAG ACG CTC TAT CTC CAA GGA CGC GGC CAG CTG CGC
  T   E   T   I   Y   Q   T   L   Y   L   Q   G   R   G   Q   L   R
 CGT GAT CTC GCG ACA GCG CTG CGT ACC GGG CGC GCA CGT CGC CGA CCG AAC
  R   D   L   A   T   A   L   R   T   G   R   A   R   R   R   P   N
 CGG GGA ACC AAC GCA CGT CGT TCG CGG TTC GTC GAC CCG ATG CTC ATG ATC
  R   G   T   N   A   R   R   S   R   F   V   D   P   M   L   M   I
 TCC GAG CGC CCC GCT GAG GTC GCT GAT CGT GCT GTT CCA GGG CAT TGG GAA
  S   E   R   P   A   E   V   A   D   R   A   V   P   G   H  [W] [E]
 GGC GAT CTC ATC ATC GGC GCT GAC CAC GCC AGC GCG ATC GGC ACC CTC GTC
  G  [D] [L]  I   I   G   A   D   H   A   S   A   I   G   T   L   V
 GAG CGA ACC ACC AGG TTC GTG ATG CTC GTC CAC CTC CCG ACC GAC CAC GCT
  E   R   T   T   R   F   V   M   L   V   H   L   P   T   D   H   A
 GCC GAG ACC GTC CGC GAT GGC CTC ATT CGG ACG ATG AGC GGC CTG CCG GCC
  A   E   T   V   R   D   G   L   I   R   T   M   S   G   L   P   A
 GAA CTG AAG AAG TCG CTG ACC TGG GAC CAG GGT GCG GAG ATG GCC GCC CAC
  E   L   K   K   S   L   T   W   D   Q  [G]  A  [E] [M] [A]  A   H
 AAG ACG TTC ACG ATC GCC ACC GAC ATG GAC GTC TAC TTC TGC GAC CCA GCC
  K   T   F   T   I   A   T   D   M   D   V   Y   F   C   D   P   A
 TCG CCC TGG CAG CGC GGT TCT AAC GAG AAC ACG AAC GGG TTG CTG AGG CAG
  S  [P]  W  [Q] [R]  G   S  [N] [E]  N   T  [N]  G   L  [L] [R]  Q
 TAC TTC CCG AAG GGC ACC GAC CTC TCA CAG CAC TCG CCC GCG GAC CTT GCC
  Y   F   P   K   G   T   D   L   S   Q   H   S   P   A   D   L   A
 CGA GTC GCA CAC GAA CTC AAC ACC CGC CCA CGC AAA ACG CTC GGC TGG GAA
  R   V   A   H   E   L   N   T   R   P   R   K   T   L   G   W   E
 ACC CCA GCC CAG CGC CTC GCT AAA CTA CCT ACC AGC TAA TCGTCGTGTTGCAACGA
  T   P   A   Q   R   L   A   K   L   P   T   S
 CCACTAGAATCCGCC
(CACCGGCCGGGCCTTTGCCG)
```

Sequence 4. IS*Lxc*3 (1,511 bp). **(a)** In between parenthesis: adjacent sequences of one copy (23° occurrence – additional file 3); **(b)** letters highlighted in green and non complementary nucleotides highlighted in yellow: IR; **(c)** letters in red: transposase start and end; **(d)** letters beneath nucleotide sequence separated in triads: transposase coding sequence; **(e)** letters within rectangles: DDE motif and other conserved amino acids of IS30 family.

IS*Lxc*4

(CAGCCGCAGCCCCCTTGACA)
GGGTGGGTTCTAACAGTGGTCGCAACACCGGCTAGTTTCGGGAGTTGTTATGACACTTGTAAGGACGTCG
CAGAAGATGACCGGGCCGGCACCGATGTCGGCCGAGCGTGCATGATATATCGAGTTGTGGCGGCAGGGA
ATG AAC ACC TCC GAG ATC TGC CGG ATG CTC GGG ATT CGC CGC AAG CTG GGC
M N T S E I C R M L G I R R K L G
AAC AAC TGG CGG AAC GGG TGG AAA CAT CGC GAC CTG GTC ACG GGC AAA GTG
N N W R N G W K H R D L V T G K V
CGT TAC TAT CCG CCG ATC GAT GAG GTT CCG GTG GTG ACG ACG GTC TCG GCC
R Y Y P P I D E V P V V T T V S A
CGG TTC TTA TCG GAG GAC GAG CGG GT**G AGG ATC GCC GAC CAA CTC AGG ACG**
R F L S E D E R V R I A D Q L R T
GGC GCG AGC ATC CGC AGT ATC GCA GCC GAC CTG GGA CGA GCA CCG TCG ACA
G A S I R S I A A D L G R A P S T
ATC AGC CGG GAA GTG CGC CGC AAT CGC GGG ATC ACT GGG G AAG TAT CGA CCG
I S R E V R R N R G I T G K Y R P
TTC CAT GCG CAC CGG CTC GCG AGG AGT CGC CGT GCC CGT GAC CGG CCG GGG
F H A H R L A R S R R A R D R P G
AAG ATC GCC TCC AAC CCC CGA TTG CAG CAG GAG ATC CGT GGG CTG TTG AAG
K I A S N P R L Q Q E I R G L L K
AAG TAT TGG AGT CCC GTT CAG ATC TGT CAG CAC CTG CGT CAG CAG CAT CCT
K Y W S P V Q I C Q H L R Q Q H P
GAC GAT CCG AGC ATG CGC GTG GTG CAT GAG ACG ATT TAC CGC GAC CTC TAC
D D P S M R V V H E T I Y R D L Y
GAC TAC CGC GGC GGC GCA CTC CCG CGC GAA CTC TGC CGA ATG CTG CGC ACC
D Y R G G A L P R E L C R M L R T
AAA CGC GAC AGA CGC AAA GCA CCC CGC GTG ATC GCC CGG CGC AGG ATC CGA
K R D R R K A P R V I A R R R I R
TTC AAC GCT GCG CTC ACG ATC CAT GAC CGT CCG TTC GCG CCC ACG GAC CGC
F N A A L T I H D R P F A P T D R
AGC GTC CCC GGA GCC TGG GAA GGC GAC CTC ATC ATG GGG CTC GGC AAC CGT
S V P G A W E G D L I M G L G N R
TCG GCG ATT GCC ACA CTC GTC GAG CGA ACG ACC CGG TTC ACT CTC CTG TTG
S A I A T L V E R T T R F T L L L
CCT GTT GAC GCT GTC AAC AGA TCC GAG AGC CTC CGC GAT CAG CTC GTC CCT
P V D A V N R S E S L R D Q L V P
GCT CTC GCG GCG CTC CCG CCC GAA CTA CGC CGC TCA ATC ACC TGG GAC CAG
A L A A L P P E L R R S I T W D Q
GGC TGG GAA ATG GCG AGA CAT GAA GAG ATC AGC CGC GCA ACA GGA ACG AGA
G W E M A R H E E I S R A T G T R
ATC TAC TCC TGC GAC CCG CAC TCG CCC TGG CAG CGC GGC AGC AAC GAG AAC
I Y S C D P H S P W Q R G S N E N
ACG AAT CGG CTC TTG CGG GAC TAC TTC CCC AAA CGC ACC GAC CTC AGA ACA
T N R L L R D Y F P K R T D L R T
CAC ACC CCG AAA GAG CTC GCC CTC GTC GCC GCC GAA CTC AAC AAC CGA CCC
H T P K E L A L V A A E L N N R P
CGC AAA ATC CTC GGC TGG AAG ACC CCG AAC ACC CTC TTC A**CT ACG CTG CTA**
R K I L G W K T P N T L F T T L L
GAA CAA ACA CAC CAT CCA TGA GTGTTGCGACCACTGTTAGAACCCACCC
E Q T H H P
(AGCCGATACAGGAACGCCGC)

Sequence 5. IS*Lxc*4 (1,311 bp version). **(a)** In between parenthesis: adjacent sequences of one copy (2º occurrence – additional file 3); **(b)** letters highlighted in green: IR; **(c)** letters in red: transposase start and end; **(d)** letters beneath nucleotide sequence separated in triads: transposase coding sequence; **(e)** letters highlighted in brown: purine rich region possible associated with +1 frameshifting; **(f)** letters in boldface: missing sequences of IS*Lxc*4 smaller version (IS*Lxc*4d1 - 896 bp long); **(g)** letters within rectangles: DDE motif and conserved amino acids of IS30 family.
