# Supplementary material for: Characterization of new IS elements and studies of their dispersion in two subspecies of Leifsonia xyli
Source: BMC Microbiol. 2008 Jul 25;8:127. doi: 10.1186/1471-2180-8-127 (PMC2516522; doi:10.1186/1471-2180-8-127)
Supplement: Additional file 3 — Mapping of Lxx (gray lines) and Lxc (white lines) IS elements within the Lxx genome based on their flanking sequences. [file 1471-2180-8-127-S3.pdf]

Additional file 3 - Mapping of *Lxx* (gray lines) and *Lxc* (white lines) IS elements within the *Lxx* genome based on their flanking sequences.

Occurrences of *Lxx* ISs [a]		Position within *Lxx* (bp)	Occurrences of *Lxc* ISs [b]		Nearby ORFs [c]	Nearby ORFs (truncated)	Within ORFs
		17,349	1	IS*1237*	Lxx00170		
		17,351	(EU599582)		-		
1	IS*Lxx*4	35,438			Lxx00340		
		36,559			-		
2	IS*Lxx*4	43,601			Lxx00410		
		44,725			-		
3	IS*Lxx*5	56,859					alpha-mannosidase
		58,353					
		116,376	2	IS*Lxc*4	-		
		116,430	(EU599583)		Lxx01180		
4	IS*Lxx*4	155,914				aryl-alcohol dehydrogenase	
		157,035			Lxx01600		
		171,771	3	IS*1237*	Lxx01750		
		171,778	(EU599584)		Lxx01760		
		181,542	4	IS*1237*			lipopolysaccharide modification acyltransferase
		181,547	(EU599585)				
		216,628	5	IS*Lxc*2	-		
		216,734	(EU599586)		Lxx02170		
5	IS*Lxx*1	227,275			Lxx02260		
		229,479			Lxx02300		
6	IS*Lxx*4	243,212			Lxx02440		

		244,333			Lxx02460		
7	IS*Lxx*2	376,418			Lxx03695		
		377,844			Lxx03710		
8	IS*Lxx*4	384,404			Lxx03780		
		385,525			-		
9	IS*Lxx*1	397,016			Lxx03920		
		399,220			Lxx03960		
10	IS*Lxx*4	417,654			Lxx04140		
		418,775				multidrug efflux membrane protein	
11	IS*Lxx*4	433,066			Lxx04280		
		434,187			(12) IS*Lxx*5		
12	IS*Lxx*5	434,614			(11) IS*Lxx*4		
		436,102			Lxx04330		
		450,313	6	IS*1237*	-		
		450,415	(EU599587)		Lxx04510		
		498,823	7	IS*1237*	Lxx05010		
		498,826	(EU599588)		-		
13	IS*Lxx*4	528,020				secreted protein	
		529,141			Lxx05260		
		530,200	8[d]	IS*Lxc*3			hypothetical protein
			(EU599589)				
14	IS*Lxx*5	546,996			Lxx05410		
		548,490			Lxx05440		
		554,002	9	IS*1237*			conserved hypothetical protein
		554,008	(EU599590)				
15	IS*Lxx*1	559,179			-		
		561,383			Lxx05560		
16	IS*Lxx*4	587,368			Lxx05780		
		588,489			Lxx05800		
17	IS*Lxx*5	605,317			Lxx05935		
		606,811			Lxx05970		

18	IS*Lxx*4	630,853			Lxx06210		
		631,974			Lxx06230		
19	IS*Lxx*4	682,147			Lxx06840		
		683,268				anthranilate synthase component I	
		724,434	10 (EU599591)	IS*1237*			phosphonoacetaldehyde hydrolase
20	IS*Lxx*4	756,491			Lxx07550		
		757,612			Lxx07580		
21	IS*Lxx*5	781,015			Lxx07755		
		782,417			-		
		842,498	11_12 (EU599592)	IS*Lxc*1			apolipoprotein N-acyltransferase
		842,503		IS*1237*			(11) IS*Lxc*1
				IS*Lxc*1			apolipoprotein N-acyltransferase
22	IS*Lxx*4	854,883					secreted hydrolase
		856,004					
		890,622	13 (EU599593)	IS*Lxc*2	-		
		890,626			Lxx08790		
23	IS*Lxx*5	961,583					two-component system, sensor protein
		963,074					
24	IS*Lxx*4	974,255					ABC-type glycine betaine transport, ATP-binding protein
		975,376					
		977,879	14[d] (EU599594)	IS*Lxc*2		ABC-type glycine betaine transport, permease protein	
		978,033	15[d] (EU599595)	IS*Lxc*2	Lxx09540		
25	IS*Lxx*5	1,028,083				proton/glutamate transport protein	
		1,029,577			Lxx10080		

		1,088,236	16	IS*Lxc3*	Lxx10600		
		1,089,312	(EU599596)		Lxx10620		
		1,156,267	17_18	IS*Lxc2*	Lxx 11300		
		1,156,310	(EU599597)	IS*1237*			(17) IS*Lxc2*
				IS*Lxc2*	Lxx11310		
		1,167,239	19	IS*Lxc4*	Lxx11360		
		1,167,301	(EU599598)	d1	Lxx11370		
26	IS*Lxx4*	1,220,958			Lxx11850		
		1,222,079			Lxx11870		
		1,278,129	20	IS*1237*			ABC-type glycine betaine transport, substrate-binding protein
		1,278,131	(EU599599)				
27	IS*Lxx4*	1,294,415					branched-chain amino acid aminotransferase
		1,295,536					
28	IS*Lxx2*	1,351,723			Lxx12960		
		1,353,149			Lxx12980		
29	IS*Lxx4*	1,399,152			Lxx13462		
		1,400,273			Lxx13465		
		1,418,384	21	IS*1237*			hypothetical protein
			(EU599600)				
30	IS*Lxx5*	1,425,659				two-component system, sensor protein	
		1,427,147			-		
31	IS*Lxx2*	1,428,339			Lxx13775		
		1,429,765			Lxx13790		
32	IS*Lxx3*	1,430,731			Lxx13800		
		1,433,367			Lxx13830		
33	IS*Lxx4*	1,473,991					potassium channel beta chain
		1,475,112					
34	IS*Lxx5*	1,489,298			Lxx14410		
		1,490,792			(35) IS*Lxx4*		

35	IS*Lxx4*	1,491,321			(34) IS*Lxx5*		
		1,492,442			Lxx14450		
		1,531,161	22	IS*Lxc4*			DNA binding protein
		1,531,162	(EU599601)	d1			
36	IS*Lxx4*	1,532,184				branched-chain amino acid aminotransferase	
		1,533,305			Lxx14750		
37	IS*Lxx5*	1,550,628			Lxx14920		
		1,552,122			Lxx14940		
		1,553,467	23	IS*Lxc3*	Lxx14940		
		1,553,479	(EU599602)			signal recognition particle, subunit SRP54	
38	IS*Lxx5*	1,644,072			Lxx15800		
		1,645,566			-		
39	IS*Lxx4*	1,665,988			Lxx16000		
		1,667,109			Lxx16020		
		1,701,440	24	IS*1237*	-		
		1,701,452	(EU599603)		Lxx16360		
		1,763,557	25	IS*Lxc2*	Lxx16970		
			(EU599604)				
40	IS*Lxx4*	1,795,008			Lxx17280		
		1,796,129			Lxx17290		
		1,807,426	26	IS*1237*			sugar ABC transporter,
			(EU599605)	d1			sugar-binding protein
		1,822,176	27	IS*1237*	Lxx17530		
		1,822,184	(EU599606)			serine protease	
		1,833,001	28_29	IS*Lxc2*	Lxx17640		
			(EU599607)	IS*1237*			(28) IS*Lxc2*
				IS*Lxc2*	Lxx17650		
		1,866,921	30	IS*1237*	Lxx18010		
		1,866,923	(EU599608)		-		
		1,917,885	31	IS*1237*		siderophore binding protein	
			(EU599609)				

		1,929,362	32	IS*Lxc*2			transcriptional regulator,
			(EU599610)				LuxR family
41	IS*Lxx*5	1,937,155			Lxx18750		
		1,938,643			Lxx18770		
		2,006,777	33	IS*1237*	-		
		2,006,927	(EU599611)	d1	Lxx19480		
		2,061,997	34	IS*Lxc*2	-		
			(EU599612)				
		2,086,080	35	IS*Lxc*3			glycosyltransferase
			(EU599613)				
42	IS*Lxx*5	2,108,451					conserved hypothetical
		2,109,945					protein
43	IS*Lxx*4	2,110,372			(42) IS*Lxx*5		
		2,111,493			Lxx20630		
44	IS*Lxx*5	2,172,361			Lxx21150		
		2,173,855			Lxx21170		
		2,189,585	36	IS*Lxc*3	Lxx21270		
			(EU599614)				
45	IS*Lxx*4	2,199,729			Lxx21360		
		2,200,850			Lxx21380		
		2,228,800	37	IS*Lxc*2	-		
			(EU599615)				
		2,228,803	38[d]	IS*Lxc*2	-		
			(EU599616)				
		2,229,709	39	IS*Lxc*3			conserved hypothetical
		2,229,814	(EU599617)				protein
46	IS*Lxx*6	2,305,178			Lxx22380		
		2,306,096			Lxx22400		
47	IS*Lxx*2	2,310,916			Lxx22520		
		2,312,342				two-component system,	
						sensor protein	
48	IS*Lxx*5	2,322,399			Lxx22678		
		2,323,893			Lxx22708		

		2,339,753	40 (EU599618)	IS*1237*		conserved hypothetical protein	
49	IS*Lxx*1	2,340,215			-		
		2,342,419			Lxx22730		
50	IS*Lxx*4	2,346,174					
		2,347,295					
		2,445,558	41 (EU599619)	IS*Lxc*3	Lxx23830		
		2,481,361	42 (EU599620)	IS*Lxc*3	Lxx24245		
51	IS*Lxx*4	2,516,751					5'-3' exonuclease
		2,517,872					
		2,546,454	43 (EU599621)	IS*Lxc*4	-		
		2,565,053	44 (EU599622)	IS*1237*			transcriptional regulator, GntR family

[a] - IS elements of the same family are indicated by color: IS5 family - green; IS481 family - red; IS30 family – blue; IS21 family - gray; IS110 – orange.

[b] - Each occurrence of IS*Lxc* is mapped on *Lxx* genome and the ID beneath are GenBank accession numbers of all flanking regions.

[c] - Ordered locus names as defined in the *Leifsonia xyli* subsp. *xyli* genome project – http://www.ncbi.nlm.nih.gov/sites/entrez?Db=genome&Cmd=ShowDetailView&TermToSearch=449.

[d] – Occurrences 8, 14, 15 and 38 of *Lxc* have only one of the flanking sequences homologous to the *Lxx* genome and the other specific for the *Lxc* genome.
