# Supplementary material for: Characterization of new IS elements and studies of their dispersion in two subspecies of Leifsonia xyli
Source: BMC Microbiol. 2008 Jul 25;8:127. doi: 10.1186/1471-2180-8-127 (PMC2516522; doi:10.1186/1471-2180-8-127)
Supplement: Additional file 4 — IS elements inserted in specific fragments of Lxc genome. [file 1471-2180-8-127-S4.pdf]

Additional file 4. IS elements inserted in specific fragments of *Lxc* genome

Occurrences of *Lxc* IS elements [a]		Nearby ORFs	Within ORFs
45 (EU599623)	IS*1237*		serine protease
46 (EU599624)	IS*Lxc*2		citrate lyase beta chain
47 (EU599625)	IS*1237*		type I restriction-modification
48 (EU599626)	IS*1237*	-	
49_50 (EU599627)	IS*Lxc*2<br>IS*1237*		hypothetical protein (49) IS*Lxc*2
51 (EU599628)	IS*1237*	-	
52 (EU599629)	IS*1237*	-	
53 (EU599630)	IS*Lxc*2	-	
54 (EU599631)	IS*Lxc*2	-	
55 (EU599632)	IS*Lxc*4d1	-	
56 (EU599633)	IS*Lxc*2	-<br>-	

[a] - IS elements of the same family are indicated by color: IS5 family - green; IS481 family - red; IS30 family – blue. The ID beneath are GenBank accession numbers of all flanking regions.
