# Supplementary material for: Characterization of new IS elements and studies of their dispersion in two subspecies of Leifsonia xyli
Source: BMC Microbiol. 2008 Jul 25;8:127. doi: 10.1186/1471-2180-8-127 (PMC2516522; doi:10.1186/1471-2180-8-127)
Supplement: Additional file 5 — Details of ISLxx6 and IS1237 sequence alignment and putative transposase amino acid sequences. [file 1471-2180-8-127-S5.pdf]

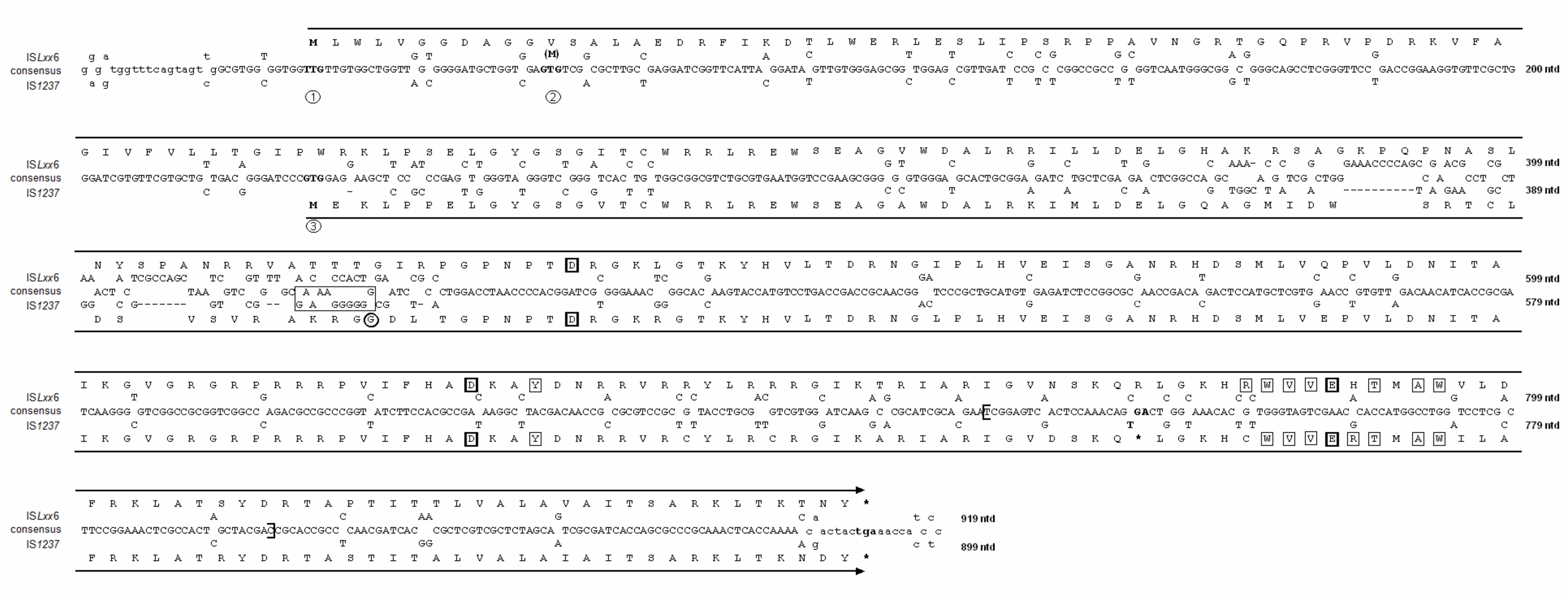

Additional file 5 - Details of IS*Lxx*6 and IS*1237* sequence alignment and putative transposase amino acid sequences. Consensus nucleotide sequence for IS*Lxx*6 and IS*1237* is presented in the same line and the divergent nucleotides are in outside lines. IRs are represented in lowercase letters and the core of IS elements are in capital letters. The amino acids for putative transposases are indicated above and below of nucleotide sequence of IS*Lxx*6 and IS*1237*, respectively. (1) and (2) indicate alternative transposase start codons within IS*Lxx*6 and (3) indicates a possible transposase start codon within IS*1237*. Nucleotides 411-421 of IS*1237* represented within a rectangle indicate the purine rich site, possibly related to a -1 frameshift. If this is the case, it promotes the translation of a glycine (within a circle) and the following amino acids. Brackets including nucleotides 706-806 of IS*1237* indicate the missing region of its smaller version (IS*1237*d1). Nucleotides 726-728 (in boldface) indicate a premature stop codon for transposase gene of IS*1237*. Conserved DDE motifs are shown within boldface squares and conserved amino acids of IS5 family are within light squares.
